# The Trend of CEACAM3 Blood Expression as Number Index of the CTCs in the Colorectal Cancer Perioperative Course

**DOI:** 10.1155/2015/931784

**Published:** 2015-10-18

**Authors:** A. Taddei, F. Castiglione, M. N. Ringressi, E. Niccolai, L. Tofani, L. Boni, P. Bechi, A. Amedei

**Affiliations:** ^1^Department of Surgery and Translational Medicine, University of Florence, 50134 Florence, Italy; ^2^Department of Experimental and Clinical Medicine, University of Florence, 50134 Florence, Italy; ^3^Clinical Trials Coordinating Center, Istituto Toscano Tumori, Azienda Ospedaliero-Universitaria Careggi, 50134 Florence, Italy; ^4^Department of Neuro-Skeletal Muscle and Sensory Organs, Interdisciplinary Internal Medicine Unit, Azienda Ospedaliero-Universitaria Careggi, 50134 Florence, Italy

## Abstract

Pathological stage seems to be the major determinant of postoperative prognosis of solid tumors, but additional prognostic determinants need to be better investigated. The most important tumor marker for colorectal cancer (CRC) is the cell-surface antigen, Carcinoembryonic Antigen (CEA), and its assessment is considered a valuable index of circulating tumor cells (CTCs). In this paper, CEACAM3 evaluation was applied given its great specificity in the CRC. Whole blood from the basilic vein of 38 CRC patients was collected before and at various time intervals after the curative resection. Also, from 20 of them, we have obtained two additional intraoperative samples. CEACAM3 expression was evaluated in all the samples by RT-PCR. CEACAM3 duct values showed a decreasing trend from preoperative through early and later postoperative to 6th-month samples (*p* < 0.001). The average values of CEACAM3 were related to the cancer size (T stage) (*p* = 0.034) and WHO stage (*p* = 0.035). A significant effect of the baseline value of CEACAM3 dCt on the temporal trend has been observed (*p* < 0.001). In this study, we have demonstrated the CEACAM3 specificity and a perioperative trend of CTCs which is coherent with the clinical/pathological considerations and with previous experimental findings in different cancer types.

## 1. Introduction

Pathological stage seems to be the major determinant of postoperative prognosis of solid tumors. This is true also for colorectal cancer (CRC) which is the second most common cause of cancer-related death in Europe and United States [1, 2]. However, within the same pathological stage, different patients may have very different disease outcomes. This remark supports the relevance of additional prognostic determinants, which are possibly linked either to the host responsiveness or to the tumor aggressiveness [3, 4]. Among the latter, in addition to vascular or perineural invasion and/or tumor differentiation and molecular characteristics, tumor markers such as the amount of circulating cancer cells/micrometastases (CTCs) may be relevant. The most important tumour marker for CRC is represented by the cell-surface antigen, Carcinoembryonic Antigen (CEA), which is encoded as well as CEACAM3 (Adhesion Molecule CEA Correlated) on chromosome 19. CEACAM3 is most specifically expressed on the cell surface of CRC patients and therefore its assessment in whole blood is considered a valuable index of circulating CRC cell number [5].

In the perspective of investigating, as a further step, potential correlation with the prognosis, the purpose of this study is the use of the CEACAM3 blood expression, as index of the amount of CTCs, to evaluate its trend at different times in the perioperative course of CRC.

## 2. Materials and Methods

The study includes 38 patients (16 females and 22 males), with a mean age of 71.0 years (range of 39–88) operated for CRC in a single institution by the same surgeon (PB) from February 2008 to July 2009 inclusively. Patients affected with cancers of the extraperitoneal rectal portion were excluded. Additional exclusion criteria were represented by metastatic disease with the exception of nodal metastases, previous chemoradiotherapy, previous/synchronous neoplasms, and immunodeficiency. Right hemicolectomy included division of the ileocolic and right colic vessels on the superior mesenteric axis; left hemicolectomy with anterior rectal resection included division of inferior mesenteric artery at its origin after identification of the hypogastric nerve.

All the patients gave their written informed consent in order to take part in the study. The study was approved by the ethical committee of our hospital. In each patient, 10 mL samples of whole blood from the basilic vein at the elbow articulation were collected each on the day before the operation, on the 1st and 5th postoperative day, and 6 months later. In 20 patients, two additional samples of whole blood were intraoperatively collected. The first was collected at the same step of the operation (just before sectioning the vein) in all the patients, in detail, from the vein draining the blood stream from the tumor (inferior mesenteric vein and ileocolic vein in neoplasms of the left colon/rectum and in neoplasms of the ascending colon, resp.). The second of the intraoperative samples was simultaneously taken from the basilic vein. Due to technical problems, only 12 of the 20 pairs of intraoperative samples could be successfully evaluated. Moreover, the results of these samples were separately considered since they are not comparable with the preoperative and postoperative ones due to the very peculiar “experimental” conditions (such as general anesthesia operative stress or different others) in which they were obtained. All the whole blood samples in EDTA were immediately stored at −80°C and evaluated for the expression of CEACAM3 by means of real-time PCR (RT-PCR) in order to assess the number of CTCs.

Control values were obtained from ten patients affected by colonic diverticular disease and comparable for age and sex distribution with patients. The controls undergoing colonoscopy, at the same time, were negative and in each of them a whole blood sample was collected and processed in exactly the same way as the study group.

### 2.1. RNA Isolation

The whole blood was defrosted. One volume of whole blood was resuspended in 1 volume of PBS and in 2 volumes of Purification Lysis Solution (Applied Biosystems, Foster City, CA). RNA was isolated using 6100 Nucleic Acid PrepStation (Applied Biosystems, Foster City, CA), according to the manufacturer's protocol, and then stored at −80°C.

The RNA concentration and purity were assessed spectrophotometrically by measuring their absorbance at 260 nm and 280 nm. RNA fragmentation state was evaluated by 1.5% agarose gel.

### 2.2. Real-Time Quantitative PCR

All RNA samples (200 ng) were reverse transcribed to cDNA using iScript cDNA Synthesis Kit (Biorad, Hercules, CA) according to the manufacturer's protocol. Negative control without RNA was performed.

TaqMan real-time quantitative PCR was performed on an ABI PRISM 7000 Sequence Detector System (Applied Biosystems, Foster City, CA). PCR products for CEA gene were detected using gene-specific primers and probes labelled with reporter day FAM (Assay on Demand, Applied Biosystems, Foster City, CA). GAPDH was used as endogenous control gene for normalization and was detected using gene-specific primers and probes labeled with reporter day VIC (Applied Biosystems, Foster City, CA).

PCR reaction was carried out in triplicate on 96-well plate with 20 *μ*L per well using 1x TaqMan Universal PCR MasterMix. After incubation for 2 min at 50°C and for 10 min at 95°C, the reaction continues for 50 cycles at 95°C for 15 sec and at 60°C for 1 min.

At the end of the reaction, the results, expressed as dCt, were evaluated using the ABI 7000 PRISM software and the Ct values were exported to Microsoft Excel.

The mean of the expression values of CEACAM3 gene obtained in controls was used as reference value to quantify the relative expression of CEACAM3 levels in CRC patients.

### 2.3. Statistical Analysis

Values are reported as mean ± SD. The assumption of normality for the distribution of the primary endpoint was tested by means of a graphical check and according to the Shapiro-Wilk test results. Comparisons of average values of CEACAM3 expression level obtained at surgery or in the postoperative phase with the baseline one were performed with Student's *t*-test for paired data. Differences in the average values of CEACAM3 expression level between different subgroups at each time in the study were tested with ANOVA or ANCOVA when adjusting by baseline value was required. The repeated measures analyses were carried out using a mixed linear model, assuming spatial power structures of the covariance matrix. All reported *p* values were two-sided. All analyses were performed by LB and LT using SAS 9.2 (SAS Institute, Cary, North Carolina).

## 3. Results

A total of 228 blood samples were collected and examined for the 38 patients enrolled in the study. Patient characteristics, site of the neoplasm, and the type of the procedure performed are given in Table 1.

### 3.1. Preoperative Findings

CEACAM3 dCt values in the whole group ranged between 2.81 and 8.35 (mean 4.01 ± 2.42). The values did not differ on account of gender, site of neoplasm, and clinical or pathological stage.

### 3.2. Findings in the First Postoperative Day

The values of the whole group in the first postoperative day ranged between −5.76 and 7.45 (mean 3.42 ± 2.69) with no significant differences (*p* = 0.06) when compared with preoperative findings. The values of the patients undergoing laparoscopic procedures ranged between −5.76 and 7.02 (mean 2.86 ± 2.51) and did not differ (*p* = 0.07) from those in the group operated with laparotomic operation (range of 0.03–7.45, mean of 4.12 ± 2.36).

### 3.3. Findings in the Fifth Postoperative Day

The values in the whole group ranged between −0.94 and 7.38 (mean 3.45 ± 2.21): differences were significant when compared to preoperative samples (*p* = 0.01). The values of the patients undergoing laparoscopic procedures did not significantly differ from those in the laparotomy group.

### 3.4. Findings 6 Months after Operation

Values ranged between −3.38 and 4.35 (mean 1.22 ± 1.62): differences were significant when compared to preoperative samples; *p* < 0.0001.

### 3.5. Intraoperative Findings

Values in the whole blood samples from basilic vein ranged between −0.93 and 5.23 (mean 1.89 ± 1.88), whereas in the samples which were simultaneously collected from the inferior mesenteric or ileocolic vein they ranged between −1.26 and 4.76 (mean 2.33 ± 1.78) with no significant differences. Intraoperative basilic vein samples were significantly higher (*p* = 0.05) than the corresponding preoperative ones. No differences were found between the “laparoscopic” and the “laparotomic” group.

### 3.6. Time Evolution of CEACAM3 dCt

**Figure 1:**
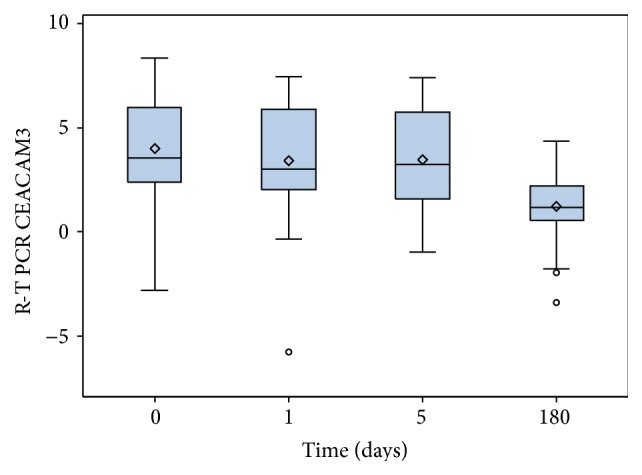
Decreasing trend of overall CEACAM3 values from preoperative through early and later postoperative to 6th-month blood samples.

**Figure 2:**
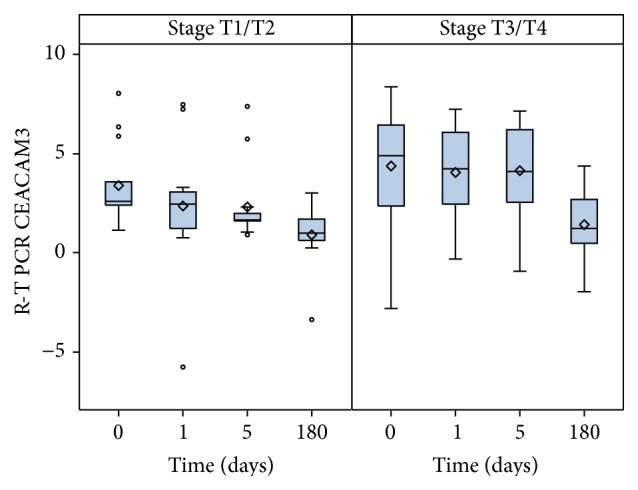
Decreasing trend of CEACAM3 from perioperative to early and late postoperative blood samples according to the pT staging.

Overall, CEACAM3 duct values showed a decreasing trend from preoperative through early and later postoperative to 6th-month samples (*p* < 0.001; Figure 1). The same trend was observed when the enrolled patients are stratified into two groups according to the pT stage (T1/T2 and T3/T4) (*p* < 0.01). Moreover, the average values of CEACAM3 duct of each perioperative set of samples (time points: 0, 1, and 6) were related to the parietal cancer invasion (*p* = 0.034, Figure 2). Meanwhile, as regards the final samples (6 months), we have observed that the values of CEACAM3 similarly decreased in both groups of patients regardless of T stage.

**Figure 3:**
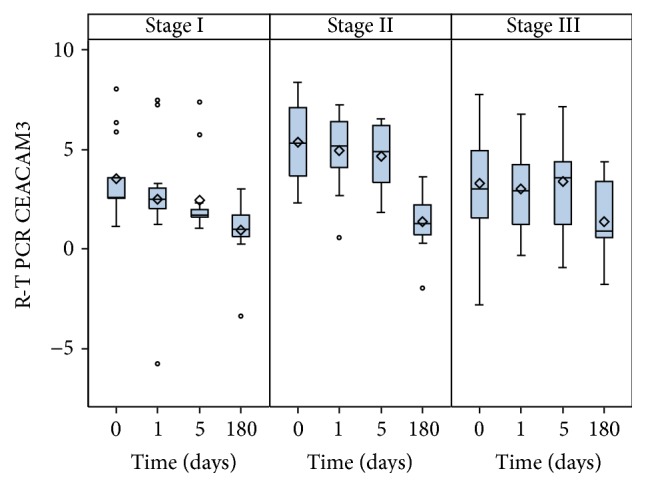
Decreasing trend of CEACAM3 from perioperative to early and late postoperative blood samples according to pathological stage (WHO).

Similar data have been observed stratifying the patients by WHO stage (*p* = 0.035, Figure 3). No evidence of any relationship between the lymph node status and CEACAM3 dCt values was found (*p* = 0.621) at any time of the study.

However, a significant effect of the baseline value of CEACAM3 dCt on the temporal trend has been observed (*p* < 0.001): a lower baseline value corresponds to a higher decrease in the 6th-month sample. Moreover, the effect of staging disappeared in the linear models adjusting by that variable (*p* = 0.075 and *p* = 0.243 for T stage and WHO stage, resp.).

**Figure 4:**
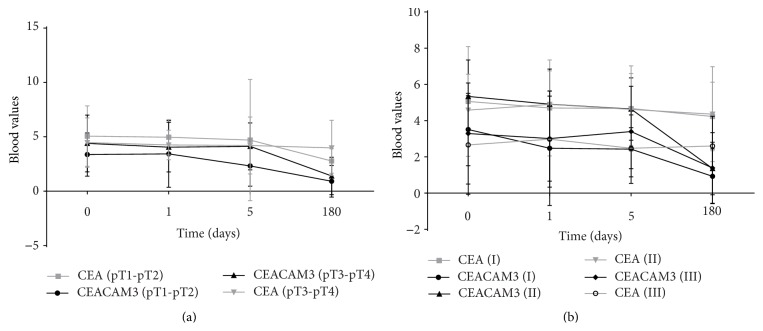
Comparison of CEACAM3 and CEA blood values in the CRC patients grouped by pT staging (a) or pathological stage (b).

Finally, to evaluate if the use of CEACAM3 as marker of CTCs is a good strategy, in the same patients, we have compared the values of CEACAM3 with blood CEA level, the most popular marker of CRC. We have observed that in both two categories (pT1-pT2 and pT3-pT4) of patients the trend of CEACAM3 is similar to that of CEA, although the average CEACAM3 values in the various time points are lower than those of CEA, suggesting a greater specificity in identifying the CTCs (Figure 4(a)). Finally, we have obtained similar data comparing the blood values of CEACAM3 and CEA in the patients grouped by the pathologic cancer stage (Figure 4(b)). Also, in this case, the values of CEACAM3 were lower than those of CEA, in particular that relating to the last blood sampling (180 days). This is very important because the value of the CEA also indicates the free antigen not bound to CTCs; on the contrary, the value of CEACAM3 essentially refers to the one linked to the CTCs and, so, is more specific marker of the CTCs.

## 4. Discussion

The novelty of this study is represented by the postoperative decreasing trend of CTCs in CRC patients. This can be considered an expected finding; however, to our knowledge, it has been demonstrated for the first time by the present paper. The CTCs are involved in the hematogenous route of metastasis and have been considered relevant for the clinical outcome of malignancies such as breast, colorectal, esophageal, and gastric cancers [6, 7]. The current definition of the “seed and soil” hypothesis is founded upon three principles. In the first place, neoplasms contain genetically diverse tumor cell subsets, each with different metastatic potential. Secondly, metastases are formed by those cells, which succeed in completing all steps of the metastatic process. Finally, the specific choice of “soil” is mostly determined by interactions between the tumor cell and the organ microenvironment. These interactions may include tumor cell specific recognition of endothelial cell antigens and response to local growth factors [8].

Since metastatic disease seems to be the most important cause of postoperative cancer-related death, detection and evaluation of CTCs could provide a deep insight in the solid neoplasm prognosis, which can be hardly foreseen on the basis of the pathological/clinical stage only. As a matter of fact, although CTCs represent a necessary (but not sufficient) condition for distant metastasis, the presence/absence and the number of CTCs may represent an important prognostic factor.

After its introduction almost 15 years ago, RT-PCR is now emerging as the most commonly used technique for the detection of CTCs [9]. The main advantage of this approach is its higher sensitivity when compared with that of other currently available methods, which utilize RNA isolation after enrichment of CTCs from whole blood [10]. To our knowledge, RT-PCR is now considered the most sensitive assay to detect tumor-specific molecular markers. Moreover, its specificity is also very high as primers are designed for the particular gene of interest and the whole genomic DNA or RNA can be analyzed in one single reaction. In the detection/evaluation of CTCs from peripheral blood of cancer patients, RT-PCR has been proposed for several genes such as CEA, PTEN, and P27 [11].

Real-time quantitative PCR of CEA mRNA methods has been previously considered reliable for this purpose [12], but CEACAM3 mRNA seems to provide, despite being at the moment insufficiently studied, the additional advantage of a greater specificity in CRC [13]. Previous data suggest that, in spite of a high sensitivity, a lack of specificity may condition the efficiency of CEA mRNA expression in detecting CTCs [14]. As a matter of fact, other causes may affect CEA mRNA: among them, the upregulation of stem cells in peripheral blood or the shedding of nonmalignant epithelial cells has been reported [15]. For this is the reason, in this study, we have used a more specific marker for CRCs such as CEACAM3.

The clinical interest in detection of CTCs is determined by its possible correlation with the prognosis. However, the purpose of the present paper is preliminary to the evaluation of the impact of CTCs on prognosis and consists of the assessment of their perioperative trend. In our study, no differences were shown between preoperative and early postoperative findings as well as within the intraoperative samples between portal and systemic blood. These data are in agreement with those previously reported and obtained with the same method and stand for a nonsignificant effect of surgical manipulation on the cancer cell shedding [16]. Moreover, they suggest that previous findings of increased postoperative levels obtained with less specific PCR methods [17] might be generically due to the shedding overall epithelial cell due to surgical trauma.

The fourfold increase in CTCs of the study group before the operation, when compared to controls, seems worthwhile to notice. This data is in agreement with previous reports in CRC, despite being obtained with different PCR methods [18]. The persistence of high values of CEACAM3 dCT up to the 5th postoperative day stands for a long life span for tumoral cells, which significantly decrease to almost normal values only in between the 5th postoperative day and the 6th month after the operation. Correspondence between the portal and systemic CEACAM3 levels suggests an aspecific filtration by the hepatic parenchyma and supports systemic levels as a representative index of the real spontaneous neoplastic cell shedding from the tumor.

## 5. Conclusions

Although the present data appear interesting, molecular and/or morphologic characterization of CTCs seem to be as important as their quantification and isolation. Therefore, possible future developments of the study will be represented by the investigation of the relationships of the different methods [19] and more specifically by the association of the present methodology with other methods of detection of CTCs such as cell search, which may allow characterization of CTCs and possibly a more accurate prognostic insight.

In spite of some limitations, the present study confirmed the specificity of CEACAM3 as marker of CCTs and showed, for the first time, a perioperative trend of CTCs which is coherent with what could be expected on the basis of clinical considerations and previous experimental findings which have been reported for both CRC and other neoplasms.

## Figures and Tables

**Table 1:** Clinical/pathological characteristics of patients and associated CEACAM3 levels.

	Time (days)	CEACAM3 levels				CEA values (Mean ± SD)
		Mean ± SD	Mean 95% CI	Median	Range	
All cases (*n* = 38)	0	4.01 ± 2.42	3.21 to 4.81	3.54	−2.81 to 8.36	4.95 ± 2.12
	1	3.43 ± 2.69	2.54 to 4.31	3.02	−5.77 to 7.45	3.93 ± 2.63
	5	3.46 ± 2.21	2.73 to 4.18	3.26	−0.95 to 7.39	4.20 ± 1.84
	180	1.22 ± 1.62	0.69 to 1.76	1.19	−3.38 to 4.36	3.80 ± 2.32
Sex						
Female (*n* = 16)	0	4.39 ± 2.34	3.14 to 5.64	4.21	1.53 to 8.36	4.98 ± 1.48
	1	3.69 ± 2.54	2.33 to 5.04	3.42	0.03 to 7.21	3.69 ± 1.82
	5	3.65 ± 2.37	2.39 to 4.91	2.56	0.89 to 7.14	3.65 ± 2.12
	180	0.97 ± 1.87	−0.03 to 1.96	0.90	−3.38 to 4.36	3.50 ± 1.65
Male (*n* = 22)	0	3.74 ± 2.50	2.63 to 4.84	3.29	−2.81 to 8.03	4.28 ± 1.89
	1	3.24 ± 2.84	1.98 to 4.50	3.02	−5.77 to 7.45	4.30 ± 2.74
	5	3.31 ± 2.13	2.37 to 4.26	3.48	−0.95 to 7.39	3.90 ± 1.65
	180	1.41 ± 1.43	0.77 to 2.04	1.25	−1.96 to 4.21	2.50 ± 1.43
Site						
Caecum/ascending (*n* = 10)	0	3.13 ± 3.17	0.86 to 5.39	2.77	−2.81 to 8.03	4.37 ± 2.35
	1	3.50 ± 2.73	1.54 to 5.45	3.16	−0.33 to 7.45	4.25 ± 1.89
	5	2.94 ± 2.72	0.99 to 4.88	2.14	−0.95 to 7.39	4.50 ± 2.38
	180	1.11 ± 1.35	0.15 to 2.08	0.83	−1.24 to 3.14	3.50 ± 2.62
Descending/sigmoid colon (*n* = 13)	0	5.06 ± 1.76	4.00 to 6.13	5.19	1.87 to 7.73	4.36 ± 1.72
	1	3.78 ± 2.39	2.34 to 5.22	4.22	0.03 to 6.74	4.20 ± 1.69
	5	4.01 ± 2.06	2.76 to 5.26	4.01	1.58 to 7.14	3.90 ± 2.15
	180	1.16 ± 2.09	−0.10 to 2.42	1.20	−3.38 to 4.36	2.50 ± 1.62
Rectum (*n* = 15)	0	3.69 ± 2.17	2.49 to 4.89	2.59	1.24 to 8.36	5.09 ± 2.33
	1	3.08 ± 3.03	1.40 to 4.76	2.89	−5.77 to 7.21	4.90 ± 2.63
	5	3.32 ± 2.00	2.21 to 4.43	3.39	1.02 to 6.51	4.90 ± 1.56
	180	1.35 ± 1.42	0.56 to 2.13	1.25	−1.96 to 4.21	3.50 ± 1.68
Stage pT						
pT1-pT2 (*n* = 14)	0	3.37 ± 1.99	2.21 to 4.52	2.59	1.12 to 8.03	5.06 ± 2.78
	1	2.36 ± 3.07	0.59 to 4.13	2.44	−5.77 to 7.45	4.96 ± 1.63
	5	2.32 ± 1.86	1.24 to 3.39	1.64	0.89 to 7.39	4.70 ± 2.58
	180	0.91 ± 1.45	0.07 to 1.75	1.01	−3.38 to 2.99	2.78 ± 1.13
pT3-pT4 (*n* = 24)	0	4.39 ± 2.61	3.29 to 5.49	4.88	−2.81 to 8.36	4.47 ± 2.27
	1	4.05 ± 2.28	3.09 to 5.02	4.23	−0.33 to 7.21	4.25 ± 1.36
	5	4.12 ± 2.16	3.21 to 5.03	4.11	−0.95 to 7.14	4.20 ± 2.62
	180	1.40 ± 1.72	0.68 to 2.13	1.23	−1.96 to 4.36	3.98 ± 2.54
Pathological stage						
I (*n* = 13)	0	3.51 ± 2.00	2.30 to 4.72	2.59	1.12 to 8.03	5.06 ± 3.03
	1	2.48 ± 3.16	0.57 to 4.39	2.47	−5.77 to 7.45	4.70 ± 2.65
	5	2.43 ± 1.89	1.28 to 3.57	1.69	1.02 to 7.39	4.67 ± 2.35
	180	0.93 ± 1.51	0.02 to 1.84	1.01	−3.38 to 2.99	4.23 ± 1.89
II (*n* = 12)	0	5.34 ± 2.01	4.06 to 6.62	5.32	2.31 to 8.36	4.58 ± 1.98
	1	4.91 ± 1.94	3.68 to 6.15	5.17	0.58 to 7.21	4.90 ± 1.85
	5	4.64 ± 1.72	3.54 to 5.73	4.90	1.84 to 6.51	4.62 ± 1.98
	180	1.36 ± 1.45	0.44 to 2.28	1.29	−1.96 to 3.60	4.36 ± 2.62
III (*n* = 13)	0	3.29 ± 2.79	1.60 to 4.97	2.99	−2.81 to 7.73	2.66 ± 2.72
	1	3.01 ± 2.35	1.59 to 4.42	2.94	−0.33 to 6.74	2.98 ± 2.65
	5	3.40 ± 2.50	1.89 to 4.91	3.56	−0.95 to 7.14	2.48 ± 1.13
	180	1.39 ± 1.95	0.21 to 2.56	0.89	−1.78 to 4.36	2.60 ± 1.64
